# Investment in plant research and development bears fruit in China

**DOI:** 10.1007/s00299-014-1587-6

**Published:** 2014-03-11

**Authors:** Kang Chong, Zhihong Xu

**Affiliations:** 1 CAS Key Laboratory of Plant Molecular Physiology, Institute of Botany, Chinese Academy of Sciences, Beijing, 100093 China; 2 College of Life Sciences, Peking University, Beijing, 100871 China

**Keywords:** Achievement of China, Plant science R&D, Plant development and hormones, Rice Biology, Plant genetics and evolution, Plant genome, Plant biotechnology, Plant protein structure biology

## Abstract

Recent rapid progress in plant science and biotechnology in China demonstrates that China’s stronger support for funding in plant research and development (R&D) has borne fruit. Chinese groups have contributed major advances in a range of fields, such as rice biology, plant hormone and developmental biology, genomics and evolution, plant genetics and epigenetics, as well as plant biotechnology. Strigolactone studies including those identifying its receptor and dissecting its complex structure and signaling are representative of the recent researches from China at the forefront of the field. These advances are attributable in large part to interdisciplinary studies among scientists from plant science, chemistry, bioinformatics, structural biology, and agronomy. The platforms provided by national facilities facilitate this collaboration. As well, efficient restructuring of the top–down organization of state programs and free exploration of scientists’ interests have accelerated achievements by Chinese researchers. Here, we provide a general outline of China’s progress in plant R&D to highlight fields in which Chinese research has made significant contributions.

## China’s research environment promotes rapid growth in plant R&D

Enough food supply is always one of the most important issues for the government in China because of its huge population. On the other hand, dwindling areas of arable lands and global climate change have challenged amount of the agriculture production. Therefore, Chinese government always has strongly supported the agriculture sciences, as well as plant sciences during the state development.

**Fig. 1:**
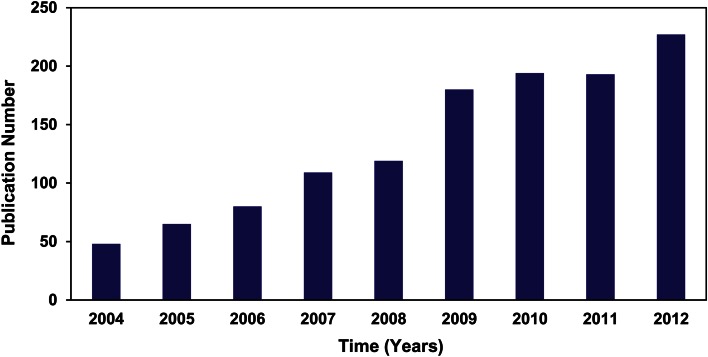
Annual plant science publications from China. Annual number of plant science publications originating from China between 2004 and 2012, based on a sample of representative journals that included Nature, Science, Cell and their sister journals, PNAS, EMBO J, The Plant Cell, Plant Physiology, The Plant Journal, PLoS Biology, PLoS Genetics and others with high impact (ISI impact factor greater than 5)

Plant molecular biology has come into a new phase in China (Chen et al. 2006), compared with the last wave, which involved plant tissue culture and its application on main crops, economically important plants, ornamental plants and tree species in the 1980–1990s. Plant research groups at institutes and universities are distributed mainly in five key areas of China: Beijing, Shanghai, Wuhan, Guangdong province and China’s western region, such as Yunnan province due to its rich biodiversity and plant resources. This distribution pattern corresponds to the one described by Prof. Tang Peisong in 1983 (Tang 1983), but there is now a larger population of scientists compared with that in 1980s. As a marker of the progress that has been made, original scientific research articles from Chinese laboratories published in international peer-reviewed journals showed a great change not only in total number but also in quality. In the past 10 years, publications in mainstream journals (those with an impact factor higher than five) from mainland Chinese groups increased more than 4.6-fold (Fig. 1). In particular, papers in the top journals such as Nature, Science, Cell and their sister journals significantly increased. Furthermore, there were more than 40 research articles from Chinese groups in The Plant Cell in 2012. The National Conference on Plant Biology, held annually, is jointly organized by the Chinese Society of Plant Biology, the Botanical Society of China, the Chinese Society of Genetics, the Chinese Society of Cell Biology, and the Chinese Society of Crop Sciences.

**Fig. 2:**
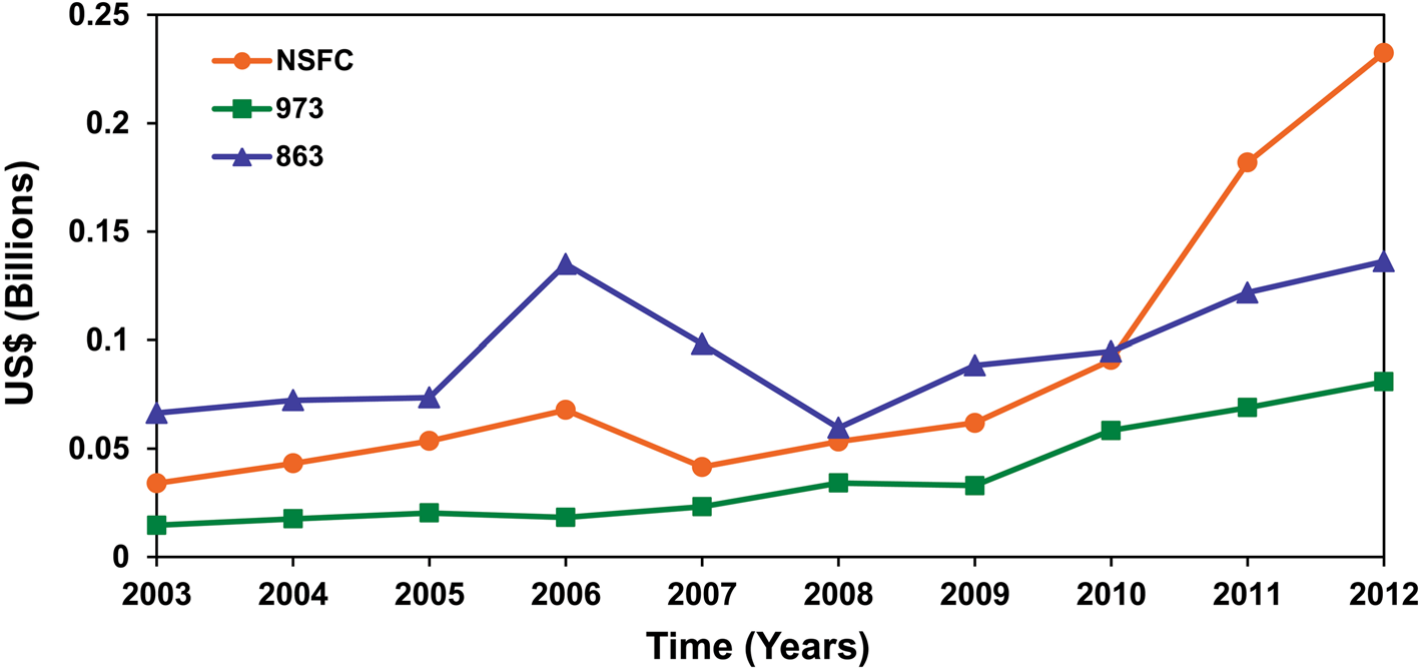
Funding trends for plant Science in China over the previous decade (2003–2012). The trends in plant science funding (exchanged into US dollars) by the NSFC, the state major basic research program (973) and the High-tech program (863)

Rice biology is a good example illustrating the speedy progress of research taking place in China. In addition to rice genome sequencing and genome-wide association studies, more than 140 agronomical important genes involved in growth and development, pest resistance and stress tolerance, especially for high-yield traits, were functionally identified (Zuo and Li 2013). More than 2/3 key genes which involved in rice grain yield and published in the top journals have been identified by Chinese groups. Another example is plant reproductive biology, for which the increase of publication numbers from Chinese groups was clearly faster than for plant biology as a whole (Fig. 2) (data from Prof Hong Ma of Fudan University). Percentage of the publication number from Chinese groups in the global total grew up to about 20 % from 7 % in the past decade. In plant biotechnology area, besides plant tissue culture, great achievements have been made in transgenic plants.

**Fig. 3:**
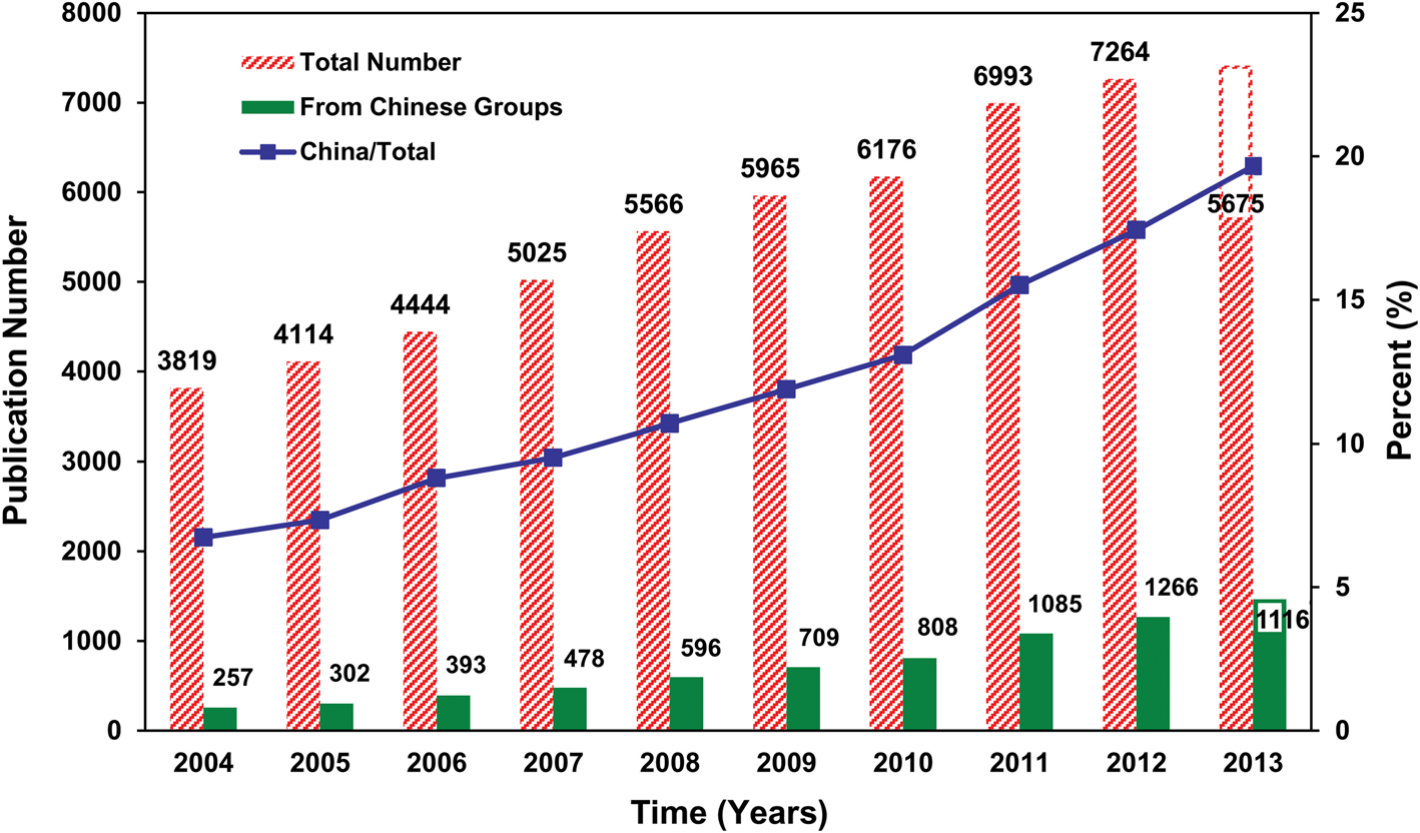
Publications in plant reproductive and development biology from China (From Dr. Hong Ma, Fudan University). Annual number of plant science publications originating from China from 2004 to 2013, based on a sample of the international journals. The dashed or hollow frame represents supposed value based on the development trends

China’s stable and high-speed economic development has supported greater funding for scientific research. The total funding for R&D reached up to 2.0 % of the Gross Domestic Product (GDP) in 2012, although percentage of funding for basic research was still lower. Plant science and its related fields have been areas of concern for the government because China perennially faces the problem of ensuring sufficient food supply to its huge population. As a Chinese economic development initiative, funding for plant R&D has been increased rapidly in the last decade (Fig. 3). The funding comes mainly from the National Natural Science Foundation of China (NSFC), the state major basic research program (973 program)and the national high-tech R&D program (863 Program) supported by the Ministry of Science and Technology, with additional R&D funding from the Chinese Academy of Sciences (CAS) and the related ministries. Notably, funding from the NSFC, which supports only basic research, has grown fivefold in the past 5 years. The NSFC has also launched a series of special major research plans, including those focusing on plant hormones (2008–2017) and genetic networks of complex traits in crops (2013–2020). Each plan has a budget of 150–200 million Chinese yuan (about 24–32 million USD). Besides, the national major R&D program for transgenic plants and animals [Phase I (1999–2005); Phase II (2007–2020)] will have total budget of 24 billion Chinese yuan (3.9 billion USD), and has promoted broad scientific research and industrialization. It includes five main crops: cotton, rice, maize, wheat, and soybean. Recently, the CAS launched a strategic priority research program (2013–2018) with designer breeding by molecular modules mainly focusing on rice and wheat. It is clear that the plant species used in the experiments have been gradually migrated from Arabidopsis to rice and other main crops, according to the national food supply needs. Overall, increasing funding from the Chinese government will continue to promote plant science and keep a balance between subject area frontiers and national needs in China.

## Genomic and genetic studies are bellwethers of rapid growth in plant science in China

Technical developments in DNA sequencing, proteomics and bioinformatics, as well as plant genetic resources including wild rice and cultivars, and T-DNA insertion lines have shed light on a range of research fields in China (Guo et al. 2006; Wan et al. 2009). Following rice genome sequencing work (Feng et al. 2002; Goff et al. 2002), Chinese groups have published a series of plant genome sequences, such as cucumber (Huang et al. 2009), Chinese cabbage (Wang et al. 2011), potato (Xu et al. 2011), *Setaria italica* (Zhang et al. 2012), *Gossypium raimondii* (Wang et al. 2012a), watermelon (Guo et al. 2012), *Citrus sinensis* (Xu et al. 2012b), *Pyrus bretschneideri* Rehd. (Wu et al. 2013), salt-tolerant plant *Thellungiella salsuginea* (Wu et al. 2012), kiwifruit (*Actinidia chinensis*) (Huang et al. 2013), etc. (some of them collaborated with foreign laboratories). Chinese scientists involved in the tomato genome consortium also participated the sequencing tomato genome (Zouine et al. 2012). Genomic and evolutionary analyses of rice, wheat, potato, cucumber, and plum blossom have been carried out (Jia et al. 2013; Jiao et al. 2012; Ling et al. 2013; Qi et al. 2013; Xiang et al. 2010). Based on genome sequences from 446 geographically diverse accessions of the wild rice species, a rice genome variation map revealed the origin and evolution of cultivated rice (Huang et al. 2012). Cucumber genomic variation map suggests one of the 112 putative domestication sweeps in these regions which contains a gene involved in the loss of bitterness in fruits, an essential domestication trait of cucumber (Qi et al. 2013). Particularly notable accomplishments include the publishing in Nature of draft genomes of wheat A (*Triticum urartu*) and D (*Aegilops tauschii*) (Jia et al. 2013; Ling et al. 2013). These draft genome sequences offer diploid references for polyploid wheat genomes, as well as providing insight into the environmental adaptation of bread wheat, and can aid in understanding the large and complicated genomes of wheat species. This progress in genome biology has been enabled by improvements in capacity for sequencing and computational developments, as well as administration and cooperation between companies and institutions (such as CAS, CAAS, universities, and BGI).

High-throughput approaches such as proteomics and transcriptomics have promoted the exploration of fundamental plant biological processes. In the early 2000s, high-throughput technical platforms based on mass spectra were established in institutions such as the Institute of Botany, the Beijing Institute of Genomics, CAS, the Beijing genomics institute (BGI), etc. Absolute quantitation of isoforms of post-translationally-modified proteins has been performed in transgenic organisms (Li et al. 2012). In the past 10 years, 160 groups from 76 institutions in China have published 280 papers on 84 species, including Arabidopsis, rice, wheat, cotton, soybean, maize, and poplar, in this research area. In particular, mechanisms of polar cell growth of pollen tubes, cotton lint and root hairs have been explored by proteomics approaches (Dai et al. 2007; Pang et al. 2010; Xu et al. 2008). Currently, proteomics research teams are working to detail networks of protein interactions including network modifications during growth and development and responses to the environment.

The state major basic research programs (973) for crops include: rice functional genomics and designer breeding by molecular modules, molecular biology of photosynthesis and biological nitrogen fixation, molecular mechanism of plant reproduction and their hormonal regulation, molecular basis of high-efficiency utilization of N and P by crops, molecular basis of pest resistance and stress tolerance, functional genomics of cotton fiber development and quality improvement, molecular biology of wood formation and tree breeding (Populus, Larix), plant secondary metabolism and molecular basis of quality improvement in main vegetables and fruits, molecular regulation of fatty acid biosynthesis in rapeseed, molecular improvement of Cassava, genetics of crop germplasms, etc.

The projects mainly focus on main cereals, such as rice, wheat, maize, and important vegetables, fruit species and woody plants. The progress has been accelerated by functional genomics of crops. For example, functional genomics projects in rice have produced a series of breakthroughs. Yield and fertility are key traits of interest for both rice production and basic research. Of rice yield studies involving seed size, seed number and tillers, the publication from Chinese groups represent about 64 % of the total published in the top journals (impact factor higher than 10). They reported that rice architecture traits (such as tillering and panicle type) affecting yield are controlled by key components such as MOC1/LS/LAS, TAD1, and TE that form a complex involved in APC/C in the cell cycle (Lin et al. 2012; Xu et al. 2012a). In addition, Li’s group demonstrated that a point mutation in OsSPL14 perturbs OsmiR156-directed regulation of OsSPL14, generating an ‘ideal’ rice plant with reduced tiller number, increased lodging resistance and enhanced grain yield. Further, Huang et al. (2009) showed that a *DEP1* dominant allele causes shorter inflorescence internodes, more grains per panicle, and a consequent increase in grain yield. Chinese groups also found that artificial selection of an amino acid substitution in PROG1 during domestication led to the transition from the wild rice plant architecture, and affected erect growth and grain yields in cultivated rice (Jin et al. 2008; Tan et al. 2008). It was also reported that the rice *GIF1* gene encoding a cell wall invertase is required for carbon partitioning during early grain filling, which is of potential use in breeding (Wang et al. 2008).

Some genetic materials have been used in traditional breeding and rice production in China, despite lack of knowledge of the underlying molecular mechanisms. In recent years, a number of such mysteries have been clarified by Chinese researchers. Chinese super hybrid rice depending on male-sterile lines has been widely used in rice production not only in China but also in other countries for decades. Molecular studies revealed that rice cytoplasmic male sterility (CMS)-related cytoplasmic–nuclear incompatibility is driven by a detrimental interaction between a newly evolved mitochondrial gene (*WA352*) and a conserved, essential nuclear gene (*Cox11*) (Luo et al. 2013; Zuo and Li 2013). Another example is photoperiod-sensitive male sterility (PSMS), which has also been used in rice breeding since the 1980s (Ding et al. 2012). As well, a killer–protector system at the S5 locus encoded by three tightly linked genes regulates fertility in *indica*-*japonica* hybrids, the information may aid in rice genetic improvement (Yang et al. 2012). The parallel-sequential divergence evolutionary genetic model in the hybrid sterility in rice involves three tightly linked loci, exemplified by a killer–protector system formed of mutations in two steps (Ouyang et al. 2011). The CSA-based photoperiod-sensitive male-sterile line allows the establishment of a stable two-line hybrid system, which promises to have a significant impact on breeding (Zhang et al. 2013).

Rapid progress has also been made in plant epigenetic research in China. Outstanding achievements have come mainly in studies of histone modification, DNA methylation and microRNAs. Specifically, protein arginine methyltransferase, SKB1/PRMT5 and its family members such as PRMT10, were found to mediate histone modification H4R3 and pre-mRNA splicing to control flowering and salt stress responses, as well as stomatal closure in response to Ca^2+^ in Arabidopsis (Deng et al. 2010; Fu et al. 2013; Wang et al. 2007). In addition, the histone methyltransferase SDG724 targets H3K36me2/3 at MADS50 and RFT1 to promote rice flowering (Sun et al. 2012). Histone demethylases that act on H3K27, such as REF6 in Arabidopsis, and on H3K4 in rice were reported to be involved in the development (Cui et al. 2013; Lu et al. 2011). Epigenome replication was found to be closely linked with DNA replication during S phase (Liu and Gong 2011), and it was revealed that the diRNAs may function as guide molecules directing chromatin modifications or the recruitment of protein complexes to DNA double-strand break sites to facilitate repair (Wei et al. 2012), representing an example of microRNAs mediating plant responses to the environment. Heat stress-induced alternative splicing was found to provide a novel mechanism for regulation of microRNA processing in Arabidopsis (Yan et al. 2012). These important works exemplify the great progress in the plant epigenetic research brought about by young Chinese groups.

There has also been a burst of high-quality Chinese papers on Arabidopsis developmental responses to environmental factors such as light, salt, and drought. Most of them have come from young scientists trained in overseas labs. For example, COP complexes were found to mediate photo- or skoto-morphogenesis. Genome-wide analysis identified transcription regulation networks involving far-red mediated hypocotyl growth (Jing et al. 2013; Ouyang et al. 2011; Tang et al. 2012). Identification of several components involved in plastid retrograde signal generation, transmission, and control of nuclear gene expression has provided significant insight into the regulatory network of plastid retrograde signaling (Chi et al. 2013; Sun et al. 2011). In terms of stress network signaling, a SOS complex and Na^+^/H^+^ antiport, as well as DNA methylation and DNA replication, were found to be involved in regulation of salt and drought responses (Gong and Zhu 2011; Ye et al. 2013; Zhou et al. 2012). It is also worth mentioning the findings that kinase CIPK23-mediated complexes and a WRKY transcriptional network (WRKY6, WRKY4, PHO1) function in nutrient stress (Chen et al. 2009; Xu et al. 2006). Phosphoinositide signaling pathway regulates multiple processes of plant growth and development, and cell responses to environmental stimuli in plants (Chen et al. 2008; Xue et al. 2009). For example, phosphatidylinositol pathway-controlled Ins(1,4,5)P(3)/Ca^2+^ levels are crucial for maintaining pollen dormancy in Arabidopsis (Wang et al. 2012a, b). Crosstalk between the phosphatidylinositol signaling pathway and auxin response is controlled by polar auxin transport (Mei et al. 2011).

In terms of photosynthesis research, China has given rise to a wave of molecular genetics, structural and computational biology studies. Representative of this is the solving of the crystal structure of a spinach major light-harvesting complex at 2.72 A resolution, revealing the first X-ray structure of LHC-II in icosahedral proteoliposome assembly at atomic detail as well as structural insights into energy regulation of LHC-II CP29 (Liu et al. 2004; Pan et al. 2011). In addition, LTD (light-harvesting chlorophyll-binding protein translocation defect) was reported to be essential for the import of light-harvesting chlorophyll-binding proteins and subsequent routing of these proteins to the chloroplast signal recognition particle-dependent pathway (Ouyang et al. 2011). Identification of several components in the plastid retrograde generation, transmission, and control of nuclear genes expression has provided significant insight into the regulatory network of plastid retrograde signaling in Arabidopsis (Chi et al. 2013).

Within plant development biology, important advances include the recognition mechanism during pollination and the genetic network underlying organogenesis (Zhang et al. 2009). Their results have provided insight into S-RNase-based self-incompatibility in flowering plants via SLF-mediated degradation (Chen and Qiu 2012; Zhang et al. 2009), the involvement of small peptides in gametophyte and pollination recognition (Liu et al. 2013; Yang et al. 2010), as well as floral organogenesis. A model of S-RNase-based self-incompatibility involving SLF-mediated degradation, based on the studies on snapdragon, was proposed to explain the biochemical mechanism for specific rejection of self-pollen tubes by the pistil (Xu et al. 2013). Pollination and cell cytoskeleton research are examples reflecting the development of cell biological research to include not only classical functional research into the cytoskeleton but also examination of novel functions of transcription factors in cellular processes involving hormone biosynthesis and signaling, such as of GAs and BRs (Li et al. 2011a; Wang et al. 2012b). In cucumber unisexual flower development, it is interesting to demonstrate at molecular level that ethylene selectively promotes female flower formation by inhibiting stamen development (Sun et al. 2010).

Although genetic and molecular networks on vernalization have been identified in Arabidopsis, cereals as well as biennial-to-perennial plants are of diverse patterns to control vernalization-required flowering. Chinese groups have shown a significant progress. An example is molecular mechanism studies on vernalization for flowering in winter wheat, which is unique, totally different from that in Arabidopsis, not only in the kinds of genes but also in network of the genes. Wheat lectin gene *VER2* could accelerate vernalization-mediated flowering (Yong et al. 2003). Lectin VER2 protein recognizing O-GlcNAc signaling on the key protein complex is involved in wheat vernalization. The findings open the way to studies of O-GlcNAc protein modification in response to environmental signals in plant development, which pattern may be shared in organisms (Lee and Shin 2009; Xing et al. 2009). Another example is that the floral transition of *Cardamine flexuosa*, a herbaceous biennial-to-perennial plant, requires vernalization. The levels of two age-regulated microRNAs, miR156 and miR172, regulate the timing of sensitivity in response to vernalization. Age and vernalization pathways coordinately regulate flowering through modulating the expression of *CfSOC1*, a flowering-promoting MADS-box gene. The related annual *Arabidopsis thaliana*, which has both vernalization and age pathways, does not possess an age-dependent vernalization response. Thus, the recruitment of age cue in response to environmental signals contributes to the evolution of life cycle in plants (Zhou et al. 2013a).

## Plant hormone researches from Chinese groups highlight the advantages

Hormone researches in China have been promoted by national research programs like the major special program on hormone function and mechanism from the NSFC and some 973 projects. These programs have attracted scientists from different fields (such as computation science, chemistry and structural biology, as well as agronomy) to solve frontier issues in hormone biology. Various hormone receptors such as COI1 for jasmonic acids (Jas), D14 complex for strigolactone (SL), and PYR/PYL/RCAR and ABAR for abscisic acid have been identified (Shang et al. 2010; Shen et al. 2006; Yan et al. 2009; Zhao et al. 2013). In addition, the newly identified hormone SL was found to function in differentiation of axillary buds in plants (Lin et al. 2009). Recently, two Nature papers from Chinese groups revealed that D14-SCF^D3^-dependent degradation of D53, as a repressor of SL signaling, regulates rice differentiation of axillary buds for tillering (Jiang et al. 2013; Zhou et al. 2013a, b). At least three aspects of China’s hormone research should be highlighted: the development of an analysis system for trace identification of hormones, the study of metabolism and signaling in plant development, and structure biological approaches revealing molecular mechanisms that underlie hormone functions.

Quantitative chemical identification of trace amounts of complex hormones has been a bottleneck hindering hormone research in past years. Now, Chinese chemists have joined the hormone research field to set up a mass spectrum (MS) system for analysis of complex hormones such as gibberellic acids (GAs) and SLs, and to create novel identification methods. For example, Feng’s group (Wuhan University) has established a MS-based method for quantitative identification of multiple GAs (up to 11 derivatives of GAs) in one sample (Li et al. 2012). In addition, a novel amperometric immunosensor for the phytohormone ABA was created based on chemical reductive growth in situ of gold nanoparticles on glassy carbon electrodes (Wang et al. 2009). Chinese labs have also produced great advances in hormone metabolism and modification, signal transduction, and hormone crosstalk, producing more than 300 publications including original papers and invited reviews in key international journals.

Structural biologists in China have collaborated with plant biologists to discover those key protein complexes in hormone signaling pathways. For instance, the structure of the ABA receptor, the PYR/PYL/RCAR complex, was resolved to clarify the mechanism of coordination between ABA and the complex (Hao et al. 2011). Structural analysis also provided insight into brassinolide perception, revealing that BAK1 is a co-receptor that recognizes the BRI1-bound brassinolide (She et al. 2011; Sun et al. 2013). In addition, the crystal structure of the two hormones signal-transducing α/β hydrolases, karrikin-signaling Kai2 and SL receptor D14 was resolved by the Xu and Li groups (Zhao et al. 2013).

## From tissue culture to transgenic crops

Research progress in plant tissue culture has been based on achievements of hormone studies from the 1970s in China. Until the 1990s, China was one of the leading countries in plant tissue culture and its applications. The tissue culture system has been approached to molecular mechanism studies on development, such as apical meristem differentiation using Arabidopsis plantlets. Using the tissue culture system, for example, it has been identified that the establishment of auxin gradients and PIN1-mediated polar auxin transport is essential for WUS induction and somatic embryogenesis in Arabidopsis (Su et al. 2009). As well, DNA methylation and histone modifications regulate de novo shoot regeneration by modulating WUS expression and auxin signaling (Li et al. 2011b). In recent decades, on the other hand, plant tissue culture techniques have been extensively used for a range of important crops and economic plants (Xu 2007). An example is micropropagation for *Eucalypyus* in Guanxi, plantlets from micropropagation tissue culture of 100 elite individual trees in the past 30 years, and annual plantlets production reaches 200 million in culture jars and 160 million provided for planting in field (Wang et al. 2007). The 2-year seed potato system ensures reduction of virus infection and hence the improvement of seed tuber quality. Virus-tested minituber of potato was applied in about 20 % potato production field in China (Liu et al. 2006). The micropropagation technique has been also used for banana, strawberry, various species and varieties of orchids in horticulture, *Dendrobium officinale* (Chinese medicinal orchid, Wu Ping, personal communication) and *Dioscorea opposite* (Li 2004), etc. Anther tissue culture has been successfully used to obtain dihaploid plants for breeding work of maize, rice, wheat and rapeseed, etc. Somatic hybrids by protoplast fusion have been used to obtain new breeding materials in wheat (Xia Guangmin’s Lab), rapeseed, potato and Citrus (Xu et al. 2012b).

Among genetic-modified crops, transgenic cotton has been planted in China for the resistance to insects as well as improvement of fiber quality. Bt insect-resistant transgenic cotton from Chinese breeders, approved in 1997 for commercial use to control cotton bollworm, has been widely used in production. In China, Bt cotton was steadily adopted by the bulk of growers (i.e., presently 95 % adoption in northern China, and 85.65 % for whole country) (Lu et al. 2010). In addition, transgenic maize harboring the phytase gene (BVLA430101) and transgenic rice with insect resistance (Huahui No. 1; Bt Xianyou 63) have obtained safety certification for production according to state regulations, although these have not been commercially planted in China. Risk assessment analyses of the transgenic crops suggest that natural refuges derived from the mixed-planting system of cotton, corn, soybean, and peanut on small-scale, single family-owned farms have a key function in delaying evolution of cotton bollworm resistance, and no trend toward Bt cotton resistance has been apparent despite intensive planting of Bt cotton in recent years (Lu et al. 2010).

Insect resistance in cotton also has been achieved by silencing a cotton bollworm P450 monooxygenase gene using plant-mediated RNAi, thereby impairing larval tolerance of gossypol (Mao et al. 2007). Another example of the improvement of cotton involves spatiotemporal manipulation of auxin biosynthesis in cotton ovule epidermal cells to enhance fiber yield and quality (Zhang et al. 2011). The lint percentage, an important component of fiber yield, is consistently higher in the transgenic plants than in non-transgenic controls, which leads to a more than 15 % increase in yield. This shows a potential for improving cotton production (Chen et al. 2010).

The Wx (waxy) locus controls amylose synthesis in rice and many other species. Wx gene expression can be regulated by antisence approach or RNAi technique in transgenic rice and cassava, which have been used to regulate starch composition. Rice endosperm has been used as bioreactor to produce HSA (human serum albumin) by transgenic rice with high expression of recombinant HAS (OsrHSA) (Yang Daichang’s lab, Wuhan University). In medicinal plant, Artemisia annua, the content of biological active component, artemisinin, is less 1 % (dry matter), but it reached 2.8 % in some strains of transgenic plants with SQSi, which has been proved for field test (Tang Kexuan’s lab, Shanghai Jiaotong University).

## Perspective

Since the new century started, the community of plant scientists has steadily enlarged in China. Correspondingly, the output of Chinese plant science research is impressive. Mainland Chinese plant researchers have published a significantly increasing share of the research articles in top journals, but breakthroughs driven by original innovations that are capable of leading new research fields and great strides in research methods are still lacking. With continuous support from the Chinese government in terms of increased funding allocations and improved infrastructure, we would like to see that Chinese plant scientists may contribute more than ever to the plant science in the world.

Using the model plant Arabidopsis, Chinese plant scientists have produced high-quality, internationally acknowledged work. However, to feed the 1.3 billon people in China and supply more food to the world despite the shrinking area of arable land, always is one of the top concerns of the Chinese government and politicians, and the responsibility of plant scientists in the world. Plant scientists need to pay more attention for staple crops such as rice, wheat, corn, soybean, rapeseed and cotton. Also, we should study those crops, like potato, sweet potato and cassava, which generally are important food resources for developing regions. When we consider to continue to increase the crop yield, we also should consider to breed more crop varieties which are environmental friendly, that means less chemical fertilizers and pesticides used. With improvement of living standard in China, Chinese scientists are to pay more attention to important vegetables and fruits for improvement of nutrient composition. For identification of active component of Chinese medicinal plants, we seriously lack the knowledge about secondary metabolism for those plants. Thus, it is important to study plant development and hormone function, plant metabolism including photosynthesis, pest resistance and stress tolerance at molecular level, and clone more functionally important genes from germplasm collection. Although many Chinese plant scientists have done impressive work in the above research areas using different crops, they should integrate their research resources, they need more interdisciplinary cooperation to solve those important plant biology problems and provide more molecular breeding approaches and to resolve major agricultural challenges. We are sure that better plant science for better life of human being.
